# Engaging with and Shaping Nature: A Nature-Based Intervention for Those with Mental Health and Behavioural Problems at the Westonbirt Arboretum in England

**DOI:** 10.3390/ijerph15102214

**Published:** 2018-10-10

**Authors:** Liz O’Brien

**Affiliations:** Forest Research, Social and Economic Research Group, Farnham, Surrey GU10 4LH, UK; liz.obrien@forestry.gsi.gov.uk; Tel.: +44-300-067-5700

**Keywords:** Westonbirt Arboretum, well-being, nature-based intervention, green mind theory, mental health, mental well-being, behaviours, behaviour change

## Abstract

Mental health problems have become one of the leading causes of disease burden worldwide. This study used qualitative mixed methods including in-situ ‘being and doing’ activities with participants, interviews, and participant observations to explore participant’s experiences of a multi-visit nature-based intervention at Westonbirt Arboretum in England. The research found that three engagement types: (1) social, (2) woodland craft, and (3) creative and sensory, provided a meaningful programme to engage those with mental health, addiction, autism and behavioural problems. These types of engagement conferred a wide range of well-being benefits on participants. The study highlights key elements of the programme that were effective including the importance of repeat visits to nature to enable familiarity, using creative, sensory and craft activities, creating a supportive environment, involving the volunteers, and understanding the needs of participants and the organisations that work with them. The research suggests that nature-based programmes can be designed to be flexible and adaptable to meet the needs of participants with mental health and behavioural problems. Small numbers of participants can be involved in an intensive and immersive way that encourages an emotional affinity with nature. Inclusive and supportive programmes are particularly important for those who are vulnerable, as they are less likely to engage with nature than the wider population.

## 1. Introduction

Mental health problems have become one of the leading causes of disease burden worldwide [1], with the main problems in the United Kingdom (UK) being depression followed by anxiety, schizophrenia and bipolar disorder [2]. Poor mental health has become an increasing source of disability in the UK with one in six people over the age of 16 having a mental health problem [2]. Poor mental health is also a contributory factor in poor physical health. People with prolonged and severe mental illness are at risk of dying on average 15–20 years earlier than other people, two thirds from avoidable physical illnesses such as heart disease and cancer, often caused by smoking [3]. The poor and disadvantaged are also disproportionately affected by common mental health problems [4]. The Mental Health Taskforce suggests the cost of mental health issues to the UK is £105 billion a year in health care costs, lost productivity, and benefit payments [3]. There is growing evidence which demonstrates a range of health and well-being benefits can be gained by individuals and groups engaging with nature across the urban–rural continuum [5,6,7]. For example, research suggests people can benefit in terms of increased attention and reduced sadness and fatigue [8,9], lower physiological stress [10] and more positive moods and emotions [11]. There is also evidence that suggests engaging with environmental enhancement schemes such as conservation volunteering can have a positive impact on identity, physical activity, psychological and social well-being [12]. Much of the research has focused on individual psychological well-being, and there has been less research on the social benefits that can be gained from nature. However, there are a number of studies that outline how nature can be a place for social interactions, whether that is via nature in residential settings, through people accessing nature for themselves, or through organised and supported interventions [6,13]. Studies have outlined benefits from interacting with nature on social cohesion [14], social empowerment [15], and social support [16]. However, social quietness and solitary connections with nature in a supportive environment can also be important for some with stress-related mental health problems [17]. An increasing number of studies have also identified the benefits of contact with nature for vulnerable groups, particularly those with mental health problems. Barton et al. [18] found that nature, exercise, and social components have a role to play in supporting those with mental health issues, others have identified that well-designed natural environments have benefits, and argue for nature as an upstream intervention to manage a range of mental health problems [19].

There is growing interest from some health professionals in nature-based interventions that can be used to support those with mental health problems or to specifically aid treatment alongside drug treatments and psychological therapies [20]. Bragg and Atkins [20] suggest that nature-based interventions can be part of solutions to support mental health care. They highlight that there are increasing numbers of local and national environmental organisations that are offering health or social care treatment interventions, environmental volunteering and care farming for vulnerable groups, such as those with mental health problems. They outline that the benefits of these interventions can include reductions in anxiety, depression and stress, and improvements in general mental well-being, self-esteem, mood, confidence, happiness, feelings of safety and security, more social contact, and feelings of being included. A key aspect of engaging with and shaping nature is also the concept of relational values; people do not only receive benefits from engaging with nature but shape it themselves through caring about nature and taking action to care for nature [21]. This perspective on caring for nature suggests that the emotional bonds between humans and nature are important and a care perspective can broaden conservation practices and utilitarian arguments about nature’s contribution to human well-being by widening the debate and highlighting the importance of human nature relations [22].

This research informs the previous work in a number of ways using a qualitative multi-method approach. It supports the existing research that highlights how targeted interventions that enable access to woodlands for vulnerable groups can have a positive impact on their well-being (mental, social, physical), and it identifies key elements of nature-based interventions that can be effective for a broad range of vulnerable groups.

### 1.1. Theoretical Context

This paper draws on the green mind theory outlined by Pretty et al. [23] which links the mind with the body and the brain. The theory suggests that environments shape people (bodies, brains and minds) and minds can change people’s behaviours and shape the external environment. The theory distinguishes between the sympathetic nervous system driving people’s ‘fight or flight’ reflex and the parasympathetic nervous system, sometimes called ‘rest and digest’. The activation of the sympathetic nervous system (SNS) increases the heart rate, blood pressure, reduces the immune function, and impacts gastrointestinal and endocrine systems [24]. The over-activation of the SNS through continual stressful situations is bad for one’s health and leads people to experience a range of feelings in response such as anxiety, fear, or anger [2]. Pretty et al. [23] argue that many aspects of modern life lead to this over-activation, however, they highlight that there are ways to quieten this reflex through engagement in activities that are immersive and focus people’s attention; thereby lowering people’s heart rate and blood pressure. Pretty et al. [23] outlines three types of engagement in particular that evidence suggests are immersive and can hold people’s attention:

- Nature engagement: activities in nature such as walking, gardening, etc.
- Social engagement: social activities that are low in material consumption such as drama groups, horticulture societies, conservation volunteering, etc.
- Craft engagement: immersive activities that hold the attention including painting, drawing, baking, carpentry, etc.

Through these types of engagement, the SNS can be quietened, enabling people to make memories, share time and experiences with others and learn new skills; all of which can impact positively on people’s health and well-being [25]. This paper uses these three types of engagement to explore a nature-based intervention and the impacts of it on people’s self-reported and observed well-being. 

### 1.2. The Westonbirt Community Project

**Figure 1:**
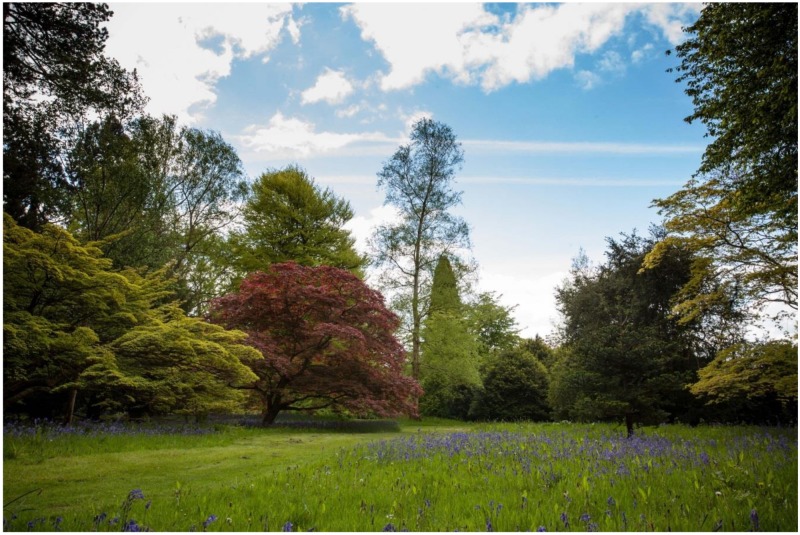
The Westonbirt Arboretum, Gloucestershire, England showing the ‘park-like’ woodland landscape.

Westonbirt Arboretum is a state forest managed by the Forestry Commission England (FCE). It is the national arboretum in England carrying out international conservation activities and is an important educational and environmental resource [26]. The site is 600 acres in size, situated in the south west of England (Figure 1). It is registered as a historic grade 1 listed park and garden, which means the site is important, distinctive, and is of special historic interest. In 2012, FCE bid for and was successful in winning Heritage Lottery funding for the Westonbirt Project. Heritage Lottery Funds are provided to conserve the UK’s heritage. The Westonbirt Project ran for 5 years from 2012–2017 and included the creation of a Community Project (CP) [26]. This paper focuses on the Community Project which aimed to increase the range of people who take part in the Arboretum’s heritage. The project focused on vulnerable (a vulnerable person can be someone who needs some sort of help with daily life due to disability, old age, social isolation, low income and who may be at risk without support.) youths (aged 13–28) and adult groups at risk of exclusion. Through the Community Project, a programme of visits was developed, including:

1. Outreach visits aimed primarily at older age groups in care homes or with dementia. Forestry Commission England staff and volunteers went beyond the Arboretum boundary directly into communities and care homes to take nature activities to those who could not easily visit the site.
2. Day visits were arranged for youth groups with autism, psychosis, and with additional needs, and older adults with dementia, and those with mental health problems and learning difficulties. Participants would spend the day at the site undertaking various activities.
3. Multi-visits involved a number of repeat trips to the site for adults with mental health problems, drug and alcohol addictions, young people with autism, and school pupils with behavioural, emotional and self-esteem problems.

This paper focuses on the multi-visits part of the Community Project to explore whether repeated trips to Westonbirt Arboretum could provide a deeper engagement with nature and an opportunity to engage and shape nature, and how this could have had an impact on participants’ sense of well-being. Specifically, the research sought to explore what type of activities the Community Project facilitated, who took part, and whether the participation led to perceived well-being outcomes for participants. The overall number of multi-visit groups and sessions are outlined in Table 1. The majority of the youth and adult groups made between 4–8 visits to Westonbirt Arboretum. A small number of schools visited every fortnight for a whole school year (September–July). Participation was free for participants as it was a funded project.

### 1.3. How the Community Project Was Delivered

**Figure 2:**
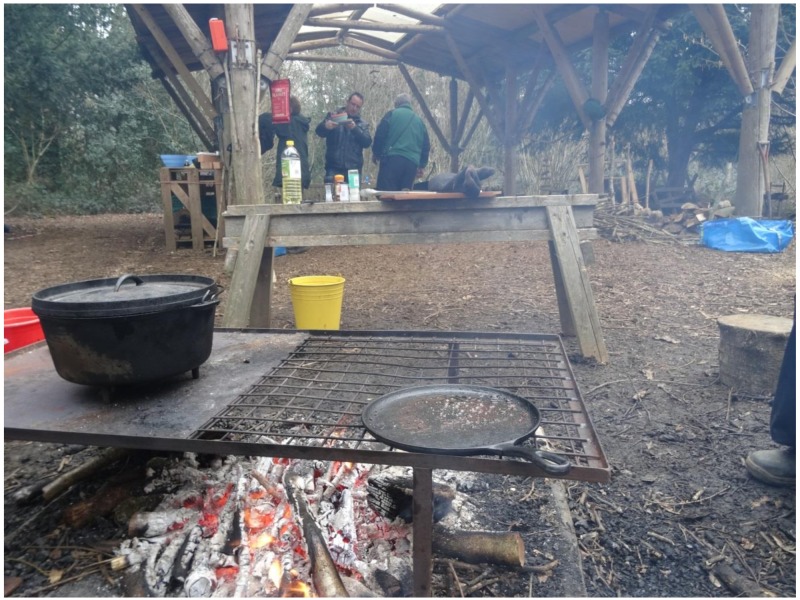
The community shelter area at Westonbirt Arboretum, showing a fire pit at the front of the picture.

**Figure 3:**
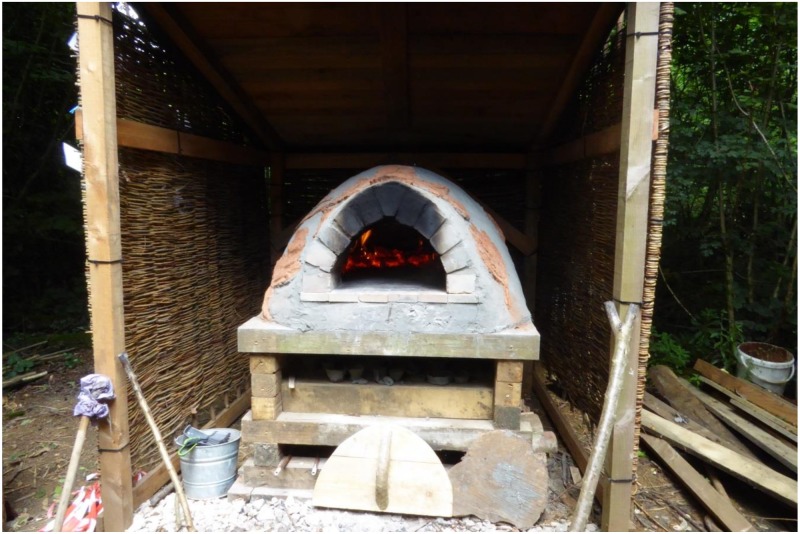
The cob oven in the community shelter area at Westonbirt Arboretum.

Forestry Commission England contacted a wide range of school, community, and residential centres within a 20-mile radius of Westonbirt to explore whether they would be interested in getting involved in the Community Project. This led to discussions about the type of participants that might get involved and the activities that could be offered by FCE. The organisations, schools, residential schools and treatment centres then identified who from their institute might benefit from the nature-based intervention; and those people were approached and asked if they want to be involved. Therefore all of the participants who agreed to participate chose to be involved. A community shelter was created (see Figure 2) away from the main public footpaths, which has a wooden structure with a roof, a table for preparing food and drinks, and some with seating, a fire pit, and a cob oven (Figure 3). Each group on their visit would be met at the Welcome Building (the main site entrance) by the FCE staff member who led the activities and FCE volunteers who supported the FCE staff, and who also worked with and helped the participants. The groups would then walk (approximately 10–15 min) to the community shelter to discuss the activities they were going to be doing that day and have a drink together. The activities and their focus were adaptable and flexible and were based on the interests and abilities of the participants. The range of activities included woodland management and maintenance such as coppicing, deer fencing, wood cutting, tree planting, and bramble clearance. There were also creative and sensory activities focusing on creating art, leaf printing, sound mapping, using taste, touch, sight, sound to explore the site, and social activities which included working with others, preparing, cooking (e.g., pizza’s in the cob oven, toasting marshmallows in the fire pit) and eating food together. Groups would spend approximately five hours on site from 10.00 a.m. until 15.00 p.m. every week or fortnight. The FCE staff who led the group activities were trained youth and community workers. An autism awareness course (with the National Autistic Society) and an inclusion training day were provided for staff and volunteers involved in the project.

## 2. Materials and Methods

The research used mixed qualitative methods to capture data in a sensitive non-threatening way from the vulnerable groups of young people and adults. The research was conducted in accordance with the code of conduct for the Government Social Research Service (GSRS, 2011) [27]. As a Government Research Institute, the members of the Forest Research’s Social and Economic Research Group (SERG) are associate members of the GSRS and follow its code of conduct. In compliance with that code, SERG has produced an ethical statement drawing on the British Sociological Association ethical guidelines, the Sociological Research Association ethical guidelines, and the Economic and Social Research Council (ESRC) research ethics framework [28]. SERG focus on informed consent, enabling participation, confidentiality and data protection, independence and impartiality, avoiding harm. Consent or assent for inclusion was given for all subjects before they participated in the study via the schools, organisations, residential schools, and treatment centres that participants were part of. The organisations’ group leaders spoke to the participants outlining what the research was about and asking for their consent or, in the case of children, asked their parents for their assent. All the data were anonymised and participants being observed were given a false name so they could not be identified by others. 

Out of the forty youth and adult groups involved in multi-visits, a sample of five youth and five adult groups were chosen to be part of the research (Table 2 and Table 3). The sample was discussed and agreed with FCE and the organisations, residential centres, and schools that were part of the intervention. The sample aimed to incorporate a range of ages, mix of gender and people with a range of different issues and problems [29]. The qualitative methods included:

1. In situ ‘being and doing’ with participants [30]: the researcher spent the day at Westonbirt for the final visit of the five youth (Table 2) and five adult groups (Table 3) and participated in the activities the groups were undertaking. The final session was chosen for the study as the groups would have experienced a wide range of activities, would have seen changes in the weather and sometimes seasons, and have become more familiar with the site over their visits and could reflect on their experiences.
2. Interviews were undertaken in situ, at the above final visits, with participants, with organisational group leaders, i.e., those who worked with participants, and who brought them to the site, and with FCE staff and FCE volunteers. Interviews were not undertaken with participants who were non-verbal or had limited verbal communication due to autism or severe learning disabilities.
3. Participant observation: FCE staff and volunteers undertook participant observation using a template and instructions developed by the researcher. Observations were undertaken at each session and focused on a sample of 2–3 people rather than the whole group (Table 4). The participants’ observed were chosen in discussion with the organisation leaders, however, sometimes a participant dropped out or could not attend some of the sessions and therefore some of the data was incomplete.
4. De-briefing sessions: after each session FCE staff and volunteers would sit down to discuss how the session had gone, any challenges or issues for participants, any changes in participant’s behaviour or enjoyment, and identify any lessons that could be learnt for future sessions with other groups. The researcher sat in on the de-brief discussions of each final visit for the five youth and five adult groups.

These methods were chosen to ensure the researcher could engage with as many types of participants as possible; including those with autism that were nonverbal. Forestry Commission England did not collect data on the number of youth or adults who dropped out of the CP. Forestry Commission England did state that a small number dropped out due to health or family reasons or clashes with other types of support the participants were receiving. Table 2 provides details on the type of youth groups and interviews undertaken and Table 3 shows the adult groups. Only one youth group included direct interviews with participants. Table 4 outlines the number of participant observations made.

The participant observation and interview methods used the ‘five ways to well-being’ framework developed by Aked and Thompson [31]. The framework was developed from a large government review of mental capital and mental health in the UK [32], which was then distilled into specific actions by the New Economic Foundation [31]. The ‘five ways to well-being’ are key actions people can take to improve their mental well-being and include:

1. Connect—to nature, people, communities
2. Be active—move around and become active
3. Take notice—of what is around you, of your surroundings, of the beautiful and inspirational
4. Keep learning—embrace new experiences, learn about nature, the community
5. Give—your time, enthusiasm, knowledge, share resources.

A template was developed by the researcher for the participant observations and was used by FCE staff and volunteers to capture data on the group name, type of group, age range, number of participants (male and female), aim of the activity, description of the activity, and location of the activity on site. FCE staff and volunteers were then asked in discussions with organisational group leaders to observe 2–3 participants at every session over their multi-visits to the site and at each session to comment on any action, discussion, comments, from participants under the ‘five ways to well-being’ headings. Table A1 provides an example of 2 visits from one young autistic group who were observed over their 6 visits to the site. Full observation notes were written up at the end of each session into an Excel spreadsheet (see Table A1).

In-situ interviews were generally fairly short from 10–20 min and had to fit in around the activities that participants were undertaking, they were digitally recorded and generally undertaken at the side of the community shelter area on a one to one basis. Recordings were transcribed. The interviews explore views on activities undertaken, perceptions of the site and the benefits gained from participation. One of the youth interviews took place with 3 young people at once. The de-brief sessions generally lasted about 20–30 min and took place after the sessions had finished, participants had left, and equipment had been put away. Field notes were taken by the researcher of the discussion at the de-brief sessions. 

### Data Analysis

All of the interviews were transcribed and imported into NVivo8 (a QSR International qualitative software package). The transcripts were read and coded for themes, which involves identifying segments of text and giving them a specific code. Top level themes were deductive i.e., the ‘five ways to well-being’ each became a top-level theme [33]. The participant observation data was written up into these top ‘five ways’ themes as well. For both sets of data, further coding was undertaken to develop sub-themes and these were then linked to the three types of engagement outlined by Pretty et al. [23]. The checklist for quality in qualitative research was followed as closely as possible: the aim of the study is outlined, along with the conceptual framework used, ethical issues are taken note of, and the methods used and coding of the data are outlined (Table 5) [34]. Quotes are used to illustrate key issues that were common across the majority of the participants and provide participant and group leaders with direct access to ways of expressing their experiences. The three types of engagement have been slightly adapted as nature engagement runs throughout the whole of the CP at Westonbirt; all of the activities took place on site and outdoors. Therefore, this paper uses the following three types of engagement, all of which take place in a natural setting:

- Social engagement: working and learning together, playing games together, preparing food, cooking and eating together.
- Woodland craft engagement: involving activities such as coppicing, deer fencing, wood cutting, log store making, tree planting, shelter creation, charcoal making, bramble clearance.
- Creative and sensory engagement: using the senses (taste, touch, sight, sound, smells), mindfulness, leaf printing, sound mapping, creating art, tasting food.

## 3. Results

The results are presented via the three types of engagement outlined above and highlight the benefits to participants related to the ‘five ways to well-being’, outlined previously. The impact of multi-visits is also presented.

### 3.1. Social Engagement

A key aspect of the CP was to enable participants to come together, work, and be together in an unthreatening way and at a rate that suited them. Participants with mental health issues, particularly psychosis, can be isolated, and learning to be and work with others is an important part of their treatment and recovery. The interviews and observations highlight that those with drug and alcohol problems can find it hard to trust others and feel comfortable with them.

*Can you imagine how isolated people are and how difficult it can be to talk to people, but here they can just naturally chat about stuff. I think people get fed up of being recognised as someone with schizophrenia sometimes they just want to be recognised as people*.

(Mental psychosis adult group, Group leader).

*I feel I’m a secluded person and do not get on too well with others but out here I feel free, no tax, no TV licence, no digital life. I feel what I’m doing at Westonbirt gets me out to socialise, I would really like to live in a wood, out in the wild*.

(Mental psychosis adult group, Male).

The CP enabled participants to step away from others when they needed to and leave technology behind; and the Westonbirt site enabled this to happen easily. The community shelter, where quite a lot of activity took place, was surrounded by trees and participants could quickly and easily step out of the shelter area into the trees to gain a feeling of being away from others while staying safe and not getting lost. For those in residential treatment and rehabilitation centres, being away from these institutional settings was viewed as very important by the participants.

*I guess we are mixing with people we wouldn’t get to know so much in our houses* [residential] *and it’s not too big a group that you feel overwhelmed and where you would struggle with interaction. It’s kind of a safe space, you feel comfortable that you can be yourself*.

(Drug and alcohol rehabilitation adult group 1, Male).

The feeling that Westonbirt was a safe place was a thread that ran through the adult groups struggling with addictions and mental health problems. This was enhanced by the FCE staff and volunteers’ approach to the CP, with participants outlining that they were encouraged in their activities, the staff and volunteers were friendly, and they were able to get to know them over time. Importantly, staff and volunteers were also non-judgemental and could be flexible in adapting the activities to suit the participant’s needs and interests.

*It doesn’t play out here, I think that is the point, we are not judged on what we’ve done or who we are or the mistakes we have made*.

(Drug and alcohol rehabilitation adult group 1, Female).

*I’ve been learning on my patience and tolerance. Tolerance of other people, getting to know people, all the people here have been really lovely*.

(Drug and alcohol rehabilitation adult group 1, Female).

Carrying out the activities at Westonbirt in the CP could also enable participants to socially interact with others outside of the sessions in a positive way by giving them something interesting and engaging to talk about, as the following quote highlights.

*Also getting home afterwards, having something nice to talk about when other people approach me about what I’m doing I don’t have to just say about the stressful things I can say I’ve been going to Westonbirt, so it helps me socially to have something nicer to talk about*.

(Supporting those with debt, addiction and mental health problems adult group, Female).

The youth groups were less likely to express their views in general. However, the observational data shows that the young people often worked together, laughed, and teased each other—mostly in a good-natured way.

*Andrew moved around the group more than normal* [in the final session]. *The whole group seemed more cohesive today. They were one group rather than lots of pairs. He floated between places, helping where he was needed, taking turns with every activity. Laughing about how tough it was to cut one piece of wood.*

(Participant observation, Youth school group 1).

A young man talked about making new friends that would continue after the CP ended. This school group was one that visited fortnightly for a whole school year.

*Yes they have put us in groups and they have mixed us up and we could speak to other people and making friends as I only knew one person in this group before I came here. Now I know everyone*.

(Youth school group 2, Male).

### 3.2. Woodland Craft Engagement

Participants carried out a range of practical tasks such as learning how to light a fire with ‘fire steels’ (two steel sticks that can be struck together to create a spark) for the fire pit and cob oven, and how to use the Kelly kettle (A device for boiling water outdoors using twigs and other combustible materials). They also got involved in woodland management and conservation activities such as fencing, coppicing, clearing bramble. They learnt to saw wood with a two-person cross saw and to split wood for the fire with a froe (a tool for cleaving wood) and a mallet. The young people particularly enjoyed it and felt they had benefited from this kinesthetic learning (learning by doing) approach.

Interviewer: What do you think you have learnt while you have been here?

*Everything, everything you wouldn’t learn in the classroom, like teachers don’t let you experiment but here they always let you have a go. They don’t just talk you through it and do it on a piece of paper they let you do it and experience it*.

(Youth school group 1 Male).

A young autistic woman was scared of fire initially, however she did try and was eventually successful in the fire lighting activity and later in the session said ‘*can I have more fire’,* she enjoyed putting more sticks on the fire in the fire pit. Adults also felt they were often doing something they had not done before.

*We talked about not being able to do the things they do at Westonbirt elsewhere—build fires, shelters etc*.

(Mental psychosis adult group, Male)

While a young man with severe autism and coordination difficulties was at first scared of using the large cross saw he was able to make progress after a number of attempts.

*Ed was very very frightened of using the saw at first and was very shaky, but he got into it and it gave him a real sense of achievement. They are developing skills. Ed has really struggled with tools*.

(Young autism group 1, Group leader).

The activities gave the young people a strong sense of achievement from not only carrying them out, but also from the physicality of the activities they undertook which could be hard work and tiring, and the contribution they were making to the maintenance and conservation of the heritage site; which became a special place for them.

*Some of it does tire you out, some of it does hurt your hands a bit (coppicing). But it’s like a nice kind of hurt. It’s a hurt you don’t mind doing, that you get a sense of achievement at the end of it*.

(Youth school group 2, Male).

These practical outdoor tasks were often things that participants had never experienced before or thought much about. However, they gave participants a real sense of satisfaction, achievement and competency for activities they often thought they could not do.

Lighting fires, coppicing, layering. I saw a tree getting chopped down over there which was pretty cool using all the old tools, which was really good. I’ve made my own pencil, made charcoal, did drawing with the charcoal. We have used the kettles and learnt how to boil our own water.

Interviewer: Are these things you have done before?

*No, never, never in my wildest dreams did I think we would do that. This is all new. In recovery you are told to find your higher power, it can be anything it doesn’t have to be God or anything like that. I love it here, I absolutely love it here, so nature is mine* (her higher power).

(Drug and alcohol rehabilitation group 2, Female).

Group leaders and the participants stated that the activities they undertook at Westonbirt were so much more than their usual idea of contact with nature; which was primarily going for a walk. Hands-on activities also allowed them to engage with and shape nature in a way many had not done previously.

### 3.3. Creative and Sensory Engagement

The creative activities included carving, making art through leaf printing, creating ‘mobiles’, i.e., sculptures that can hang from a tree or fixture. It also included the preparing, making and cooking (in the cob oven) of food that participants eat for their lunch. Pizzas were most often made and participants could be creative in what they added to their pizza and what shape it was. Sensory engagement gave participants time to take notice of what was around them and could involve mindfulness sessions were participants closed their eyes and listened to what they could hear in the forest. Walks and activities around the site could involve feeling the bark of trees, tasting different things such as flowers and leaves, as well as seeing the sights of the Arboretum and experiencing changes in the weather and seasons. Poor weather was disliked by some, however, for others, it was viewed as a part of the whole experience. Making food ‘off the grid’ gave a sense of achievement.

*It’s quiet, you’re not relying on technology, it’s back to basics. There is no outside interference it’s just nature. Being able to start a fire without a lighter and make pizza without a gas oven. It gives a sense of satisfaction*.

(Drug and alcohol rehabilitation group 2 Male).

Spending time listening to the sounds in the wood gave participants an opportunity to be calm and to quieten their mind.

*My favourite thing was the mindfulness. It’s sitting with your mind, you have things going through your mind 24 h a day right but with mindfulness it’s about you being in control, sitting there and seeing what’s in your mind but not letting it take over and just letting things pass through. You can do breathing techniques as well to regulate the body. So we walked through the other side of the arboretum up through the redwoods and we all found a bit of time to go off and sit by ourselves so I chose a tree and sat down next to it put my jacket over my knees like a little old lady but I don’t care and the sun was shining down on me and literally you hear the birds, I’ve never really sat and listened to the birds lost in that tranquil moment. It felt really calm and safe*.

(Drug and alcohol rehabilitation adult group 1, Female).

*He talked about enjoying being out and how it’s peaceful and an escape from chaos. How it gives him time to think and reflect*.

(Participant observation, Drug and alcohol rehabilitation group 2, Male).

Creative activities could be tailored for all ages and abilities so that participants could achieve making something they probably had never made before.

*Amelia was interested in creative activities, such as making a pencil, ink, a notebook, an origami bird. She was making sure she was working correctly*.

(Participant observation field notes, Youth school group 2).

Different parts of the site could also foster creative and sensory engagement. Westonbirt in recent years had a treetop walkway built; this 300m walkway is step free so accessible to those with buggies or in wheelchairs, or those with mobility issues. It rises above a valley into the trees, has interpretation features to engage with, and a crow’s nest lookout point that people can climb up to. It was not funded through the Heritage Lottery Project but is proving very popular with the majority of visitors to the site. The CP participants had to walk over the treetop walkway to reach their community shelter.

*Jack remembered that we would be walking over the treetop walkway and was very excited and keen to get there. He walked over the rope bridge, climbed the steps to look out from the crow’s nest and engaged with many of the interpretation features along the way*.

(Participant observation field notes, Young autism group 1).

The creative and sensory activities were often ones that participants did not often undertake and they were viewed as different and interesting by them, and carrying out these activities outside in beautiful surroundings was particularly novel. Working at some of the creative and practical tasks with others suffering from similar issues and problems also gave many of the participants a sense not only of achievement, but of worth.

*Yes, it’s been great, it’s a bit different from what I thought. I was never into trees in the first place. But it’s been good as there have been different aspects–doing the tasting, the sound and the willow making. It makes a change from lounging on the sofa cause no one comes to see you. This is different you get out in the fresh air*.

(Social prescribing group, visually impaired Male).

*There is not one person here who in the last 6 weeks has not had one of those moments to be at peace, to feel I am creative, I am worthwhile*.

(Drug and alcohol rehabilitation, group 2, Group leader).

### 3.4. The Importance of Repeat Visits

The repeat visits were particularly important for these participants as they were facing a range of addiction, mental health, and behavioural problems. Participants often spoke about feeling nervous on their first visit, unsure of what they would be doing and what was expected of them. This was because the majority did not know the site and were unsure of how they would cope, and they did not always know the other participants. All of this created anxiety for people who were already facing a number of challenges. The repeat visits allowed participants time to get to know both the place and each other better, to build trust and develop relationships, and engage in activities they could practice over time at each visit in varied combinations to persevere, reinforce learning, and promote confidence.

*She seemed positive about the session and told me it was a shame it was the last one as she would have liked more and felt she had made friends now*.

(Participant observation. Supporting those with debt, addiction and mental health problems adult group, Female).

*Always friendly but concentration very variable, particularly if he thinks he will not be good at a task. Over the year his concentration and self-esteem have improved*.

(Participant observation, Youth School group 2 Male).

Some participants felt that they would have liked to come for a longer period than 4–8 visits, to really get to know the site and the activities they were undertaking in more detail. Others wanted to do the whole intervention again; however, the funding was not able to cover this.

The size of the groups was generally small (with 3–10 people), and there was a high level of support for participants as an FCE staff member led the sessions and was often assisted by one or two volunteers. The organisational group leaders who brought the groups to the site would also participate in the activities and support the participants. The group leader was a link to their everyday situation and was familiar with the problems and issues they faced. Over time, some of the young people who participated for the whole school year could also see the results of their activities:

*We have been doing the coppicing just back there and they do see the difference they make and they do the deer fencing and know why it is important. It is hard physical work sometimes*.

(Youth Group 2, Group leader).

*I think 6 weeks is a good period of time to be doing this. It would be great if it could be longer and I’m sure the clients would love to keep coming*.

(Drug and alcohol rehabilitation group 2, Group leader).

## 4. Discussion

### 4.1. The Three Types of Engagement

The evidence from this study suggests that the coming together of the three types of engagement (social, woodland craft, creative, and sensory) adapted from Pretty et al. [23] were particularly important in combination over the multi-visits that participants made to Westonbirt Arboretum [35]. Each type of engagement was considered by FCE staff in discussion with organisational group leaders, and how the engagement could be used to provide the varied groups with activities that were interesting, with some challenge and where they could learn a skill, or learn about themselves. The activities were meaningful as they were making and doing things with their hands, using their bodies and muscles to create, conserving and shaping nature, which also facilitated memorable experiences; identified by others such as Lovell et al. [12], Bragg and Atkins [20], and Townsend [36]. The activities did allow for the immersive-attention outlined by Pretty et al. [23] that can quieten internal mental chatter. The youth groups were particularly engaged with the woodland craft activity, the excitement of being able to light a fire and to use a saw and mallet, as these were things they would never normally be allowed to do. However, it was clear that some of the young autistic people feared some of the activities at first, potentially because they were so unfamiliar. They were encouraged and supported and discovered that they could persevere and overcome some of their fears. The adults particularly engaged with the creative and sensory activities allowing space and time to feel calmer, be away in a very different environment and become absorbed in their activity. This fits well with Bragg and Atkins [20] and others’ findings that the three key components of nature-based interventions for mental health care are (1) the natural environment, (2) meaningful activities [37,38], and (3) social context [35].

### 4.2. The Five Ways to Well-Being

The five ways to well-being was a useful framework for data gathering. It is important to note that there are many linkages between the different types of well-being. For example, the ‘mental’ well-being gained by some participants by being away from their usual environment was linked to the mindfulness activities they undertook when they were focusing on ‘taking notice’ of their surroundings, and of the sounds and views in the forest. ‘Learning’ and skills development benefits could be linked to the ‘physical’ activities such as coppicing and fencing that participants were undertaking and enjoying, which gave them a satisfied tired feeling at the end of the day. The concept of ‘giving/sharing’ was closely linked to ‘connecting with other people’, as participants shared their enthusiasm and experiences with others. While ‘taking notice’ was also closely linked to ‘connecting with nature’ as participants took time to explore what was around them and understand more about the trees and wildlife in the Arboretum.

### 4.3. The Role of Nature in the Nature-Based Intervention

The Westonbirt Arboretum site was the location and the focus of the activities participants got involved in. As the national arboretum with a world class tree collection and as a historic grade 1 listed landscape, Westonbirt is a special landscape that receives four hundred thousand visits per year. The youth and adult participants, most of whom had not visited the site previously, were able to spend time in this special landscape, and in an area that was specifically created for them (the community shelter). The participants felt a sense of ownership of the community shelter where they spent some of their time on most of their visits. They were able to experience a site with many more trees of all species and sizes than they would normally come across. The size of the site provided an experience of majesty and freedom and a picturesque setting [6,39]. Being away from their clinical and residential or school setting was also very important in enabling this sense of freedom and release, and having a space to leave behind some of the worries, problems and intensities of their situation and treatment [39,40]. Participants talked about the atmosphere (calming, peaceful, stress-free, tranquil) and beauty of the site and the size of the site provided feelings of spaciousness with opportunities to explore, see, and experience new things at each visit; aspects that have also been found in other research [41,42,43,44,45].

### 4.4. Implications for Management and Legacy

Forestry Commission England staff played a key role in developing and facilitating the CP; they engaged with the organisations that brought participants to the site and were able to adapt the approaches taken based on the needs of participants and suggestions from the various organisations. The FCE staff also actively engaged volunteers in the CP. Volunteers importantly supported both staff and participants, with some volunteers developing strong relationships with the participants over time. Both FCE staff and volunteers collected participant observation data which took extra time and commitment that needed to be factored into the CP, and which needed senior management support. Using a public forest site open to all means that the participants and organisations can come back to the site after their engagement with the CP ends. Creating a specific space (the community shelter) for use by participants provided them with a sense of ownership and a space that they could feel was safe and which was away from the majority of the public visitors. Many projects end once specific funding finishes, as project leaders move on to find other work, and organisations often struggle to keep the momentum going. However, the CP at Westonbirt does have a legacy; FCE took a strategic management decision to continue work in this area and made the Community Project officer post permanent. Forestry Commission England is also developing a community access scheme to support local groups and organisations that work with people with mental health problems and disabilities to help them visit the site at a reduced cost. A book has also been produced by FCE staff to provide a resource that champions the importance of engaging with vulnerable community groups [46]. Training is important and a staff member is training as a Dementia Champion and will help roll out Dementia awareness to other staff and volunteers across the site. 

### 4.5. Research Challenges

A criticism by Bragg and Atkin [20] related to nature-based interventions for mental health care is that there is a lack of coordination; organisations do not always come together to collaborate across the sector and provide a more coherent joint offer to mental health services or take a common approach to evidence gathering. Most often interventions are researched and evaluated using different approaches so that comparisons across interventions are problematic. Longer term and larger scale research is needed, but rarely funded, to understand how nature-based interventions compare with other interventions and treatments, and to understand what, if anything, is important about the contribution of nature. Greater collaboration is needed across the environment and health sectors to explore these issues and develop and agree on some common metrics and indicators for use in evaluations and research.

### 4.6. Limitations of the Research

The research was limited to a single site with no control or comparison. It would have been useful to explore and compare some of the other non-nature activities participants undertook in their residential settings in order to explore the role and inclusion of nature as part of their rehabilitation or treatment process. However, the funding did not allow for this scale of research to be undertaken. More interviews were undertaken with adults than with school pupils. This was due to a number of the young people who were autistic with limited speech. Therefore the views of group leaders were used to explore the impact of engagement with nature on the young people. Interviews were generally short and we, therefore, used other methods to ensure there was enough data to report on.

## 5. Conclusions

Longer term engagement with nature is important [47,48], it can be especially significant for those who are excluded and who have diverse mental health conditions [49], as these are people who are less likely to engage with nature in the UK and gain the benefits outlined in much of the recent research [5,6]. A World Health Organisation [50] report on urban nature-based interventions outlines the importance of planning and designing interventions with the specific community intended for involvement in mind. This was the case with the CP at Westonbirt. The fact that many of the organisations working with FCE wish to continue their involvement is positive shows that these organisations have seen a benefit to their participants and can envisage more of their clients benefiting from it [44]. Westonbirt Arboretum was a non-clinical or residential setting for participants that allowed for the three types of engagement to take place. The structured and creative approach allowed for flexibility and could be adapted and changed to meet the diverse needs of participants. Increasingly, nature-based interventions such as this, are being used to support health and social care services and can have an important and positive role to play in contributing to the well-being of vulnerable young people and adults.

## Figures and Tables

**Table 1:** The total number of youth and adult groups involved in the Community Project multi-visits and the number of sessions they were involved in.

	Youth Groups (Number of Groups Involved)	Youth Sessions (Number of Sessions Participated in by the 22 Youth Groups)	Adult Groups (Number of Groups Involved)	Adults Sessions (Number of Sessions Participated in by the 18 Adult Groups)
Multi-visits	22	221	18	91

**Table 2:** The five youth groups involved in the research.

Group	Situation and Context	Interview with Group Leader	Interview with Participants	Interview with FCE Staff	Interview with FCE Volunteer
Young autism group 1 (aged 16–17)	In a residential school. 3 young men on pre-work experience at a residential school for those with severe learning disabilities.	1	0 (3 ^1^ young men participated)	1	1
Young school group 1 (aged 13–14)	State school. Behavioural problems, low self-esteem. Vulnerable young men who find it difficult to cope in an educational setting 5 days a week.	1	0 (8 young men participated)	1	1
In residential accommodation. Severe autism. Nine carers came with ten young people. Many of the young people were non-verbal.	Young autism group 2 (aged 17–19)	2	0 (10 youth participated)	2	1
Young autism group 3 (aged 18–28)	Not residential group living at home.	1	0 (1 female, 2 males)	1	2
Young school group 2 (aged 13–14)	Identified by the school as low self-esteem and confidence.	1	5 (2 females, 3 males)	0	3
**Total number of interviews (24)**		**6**	**5**	**5**	**8**

^1^ In brackets is the numbers involved in the session rather than the number interviewed.

**Table 3:** The five adults groups involved in the research. FCE = Forestry Commission England.

Group	Situation and Issues	Interview with Group Leader	Interview with Participants	Interview with FCE Staff	Interview with FCE Volunteer
Mental psychosis group (aged 25+)	Residential treatment centre. Early intervention for mental psychosis.	2	6 (1 female, 5 males)	0	0
Drug and alcohol rehabilitation group 1 (aged 30–55)	Residential treatment centre for addiction problems.	1	8 (5 females, 3 males)	1	3
Supporting those with debt, addiction and mental health problems (aged 30–60)	Socially excluded and vulnerable suffering from mental and physical health issues, many on low incomes or benefits.	2	2 (1 male, 1 female)	2	1
Drug and alcohol rehabilitation group 2 (aged 30–55)	Residential treatment. Addiction problems	1	4 (1 female, 3 males)	0	0
Social prescribing group (aged 20–60)	People with low well-being and social isolation.	1	4 (3 female, 1 male)	0	0
**Total number of interviews (38)**		**7**	**24**	**3**	**4**

**Table 4:** The number of participant observation comments made.

Groups	Number of Participant Observation Comments
5 Youth groups	526
5 Adult group	453

Participant observations were made at all of the sessions of the five youth and five adult groups. In each session, a single person might have 5–7 observational comments made about them. This included a comment on each of the ‘five ways to well-being’ (see below), and sometimes an overall comment, and any comments or reflections the group leader made. Therefore over, for example, 5 multi-visits, a single person might have 25–35 observations made of them).

**Table 5:** The data coding structure using the five ways to well-being as a deductive framing and with sub-themes developed inductively.

Higher Tier Themes—Five Ways to Well-being (Deductive)	Lower Tier—Sub-Themes (Inductive)	Links to Three Types of Engagement
Connect—people	Trust	The three types of engagement: Social engagement Woodland craft engagement Creative and sensory engagement could be linked to all of the five ways to well-being. Social engagement was particularly linked to connect with people and share/give.
	Supportive atmosphere	
	Openness	
	Safe	
Share/Give	Experiences	
	Enthusiasm	
Connect—nature	Sensory stimulation	
	Being away	
	Emotional connection	
	Sense of place	
Keep learning	Achievement	
	Learning by doing	
	Perseverance	
	Learn about oneself	
Take notice	Weather/seasons	
	Trees and wildlife	
	Peace and calm	
Be active	Physical movement	
	Practical meaningful activities	

## Supplementary Materials

The following report is available at https://www.forestresearch.gov.uk/research/evaluation-of-the-westonbirt-arboretum-community-inclusion-programme-and-visitor-experience/.

## Appendix A

**Table A1:** The example of data in an Excel spreadsheet from participant observations made by the FCE staff and volunteers. The names of the participants and data gatherers are changed. Data were collected under the ‘five ways to well-being’ themes along with an overall comment.

Group:	Young Autism Group 2	
**Description of group**	Group of young people post 16 Vocational provision	
**Data type: (e.g., community programme group)**	Community programme group	
**Age range of group:**	17–19	
**Has verbal permission been sought from participants for being involved in the evaluation—Yes/No**	Yes—from support workers and lead at school as not all participants are verbal	
**Data: Weekly Comments**	Week 1	Week 2
**Number in the group on the day**	9 participants and 8 support workers	8 participants, and 7 support workers
**Number of men and women**	6 young men 3 young women	6 young men 2 young women
**Data entry by:**	AB	AB
**Description of activity**	Sensory Walk, Play trail, nature printing, scavenger hunt	Target setting, xylophone making using saws, measuring, drilling holes, pairs matching game, sensory sounds, touches, smells, Tyre tunnel and bird viewing area.
**Aim/purpose of activity**	exploring with senses, becoming accustomed and aware of Westonbirt as a place	Exploring with senses, observing wildlife, learning safe tool use, confidence building.
**Location of activity**	Learning Centre and Old Arboretum	Learning Centre and Old Arboretum
**Proxy name: Polly**	**Date of session: 09.06.16**	**Date of session: 16.06.16**
**Overall comments about the person during the session**	Took part in every activity. Polly is quite emotionally literate and comfortable talking to others.	
**Take Notice**	Asked questions about leaf printing.	Said she wanted to learn about bird names.
**Share/Give**	Took turns with the pad.	Asked if another participant would like a turn threading rope through the xylophone and said. “I’m letting Kevin have a turn, teamwork!”
**Connect with nature**	Looked at leaves while nature printing and at the arboretum while on a walk.	Asked about moss on a hazel log “is that called moss?” Showed pleasure while watching birds in the bird hide.
**Connect with people**	Talked to support workers and our staff and volunteers about activities.	Laughing at others’ results during the pair game. Made a joke about mushrooms (there was a picture of a mushroom on one of the wooden discs she “won”). Talked to the volunteer and enjoyed filling in her target sheet with help.
**Learn**	Learnt some names of trees while leaf printing. When first walked along the balance log, she got a bit scared and did not get to the end. Walked back along a second time, this time completing it.	Learned the names of some birds. Discussed the need to wear gloves while sawing to avoid hurting fingers. Wrote on target sheet that she had learnt the words saw, xylophone, drill.
**Be active**	Walked on the balance log in the victory glade and walked through the old arboretum.	Walked to the bird hide and back. Ran back from the toilet as it was raining.
